# Building upon the foundational science curriculum with physiology-based grand rounds: a multi-institutional program evaluation

**DOI:** 10.1080/10872981.2021.1937908

**Published:** 2021-06-11

**Authors:** Arielle L. Langer, Brian L. Block, Richard M. Schwartzstein, Jeremy B. Richards

**Affiliations:** a Division of Hematology, Brigham and Women’s Hospital, Boston, MA, USA; b Division of Pulmonary, Critical Care, Allergy and Sleep Medicine, University of California San Francisco, San Francisco, CA, USA; c Division of Pulmonary, Critical Care, and Sleep Medicine, Beth Israel Deaconess Medical Center, Boston, MA, USA

**Keywords:** Mixed-learner environment, near-peer teaching, physiology, clinical reasoning, concept map

## Abstract

**Introduction**: Vertically integrating physiology into patient care has the potential to improve clinical reasoning. Clinical Physiology Grand Rounds (CPGR) is a case-based teaching method that brings together students from all years of medical school to focus on linking clinical presentations to core basic science concepts including anatomy, physiology, and pathophysiology. In this study, we describe the implementation of CPGR at two different institutions in the United States and assess student-reported outcomes.

**Methods**: We survey students who participated in CPGR at Columbia University College of Physicians & Surgeons (P&S) and Medical University of South Carolina (MUSC). Subjects were queried across three domains: the benefits of attending, the impact of concept maps, and the impact of the mixed-learner environment.

**Results**: Despite differences in session leadership and the underlying medical school curricula, conference attendees reported similar benefits at the two schools included in this study. Students overwhelmingly (92.9%) reported that remembering clinical presentations was easier when they understood the underlying physiology. They also reported gaining a true understanding of concepts that were previously memorized (87.5%). Both clinical (92.5%) and preclinical students (93.1%) valued the mixed-learner environment as a component of the conference.

**Discussion**: By assuring a mixed-learner environment with near-peer interactions, using concept maps as a teaching tool, and rigorously linking clinical presentation and management to physiological concepts, we found that the key benefits of CPGR were replicable across different institutions, despite several local differences in how CPGR was implemented, led, and conducted.

## Introduction

Medical students often find the transition from the classroom to the wards daunting [1]. Teaching strategies that do not link the preclinical and clinical curricula can exacerbate this impression by creating the false perception that preclinical coursework does not directly relate to patient care and miss important opportunities to reinforce and build upon foundational concepts in medicine. Furthermore, there are few validated approaches to explicitly and effectively connect these realms of learning, and students often fail to recognize how much they can learn from their peers and near-peers in both clinical and preclinical settings [2].

**Figure 1.**
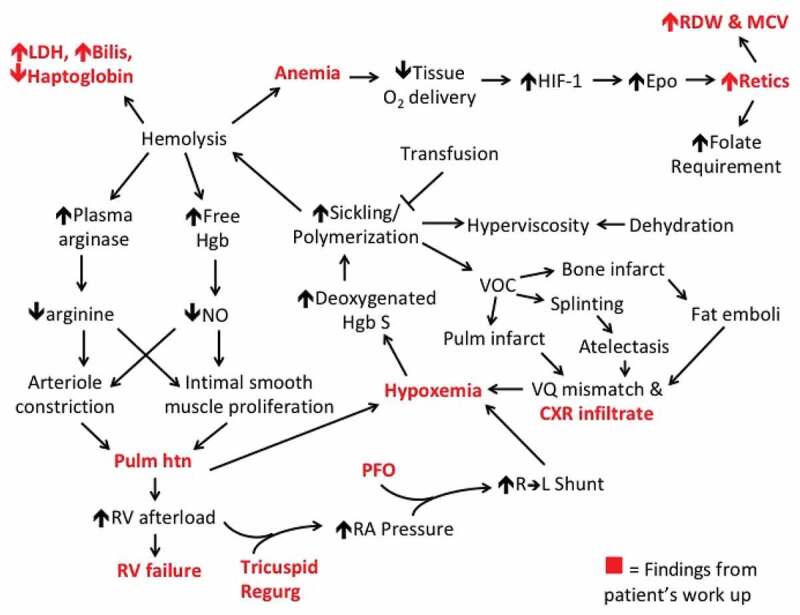
Example Concept Map (CM)

Clinical Physiology Grand Rounds (CPGR) was created to explicitly address these deficits by vertically integrating preclinical coursework with clinical learning and utilizing near-peer interactions [3]. Originally developed at Beth Israel Deaconess Medical Center (BIDMC) and Harvard Medical School (HMS), CPGR is an interactive presentation and dissection of a clinical case. A senior student presents the case, faculty facilitate the discussion, and audience members – students from all four years of medical school – interpret the information as it is presented, with assistance from faculty. In the process, faculty and students create concept maps [4] (Figure 1), which are developed and utilized in real-time. These concept maps are used to prompt students to be rigorous in relating clinical presentations to underlying physiology, thereby emphasizing the link between clinical and preclinical learning.

This concept map (CM) relates the findings of a patient with sickle cell disease complicated by pulmonary hypertension presenting with a vasocclusive crisis (VOC) that evolved into acute chest syndrome.

As detailed previously [3], CPGR leverages several evidence-based teaching modalities to effectively engage students. The mixed-learner environment fosters student interaction and near-peer teaching. Use of the Socratic method, with reliance on questions that being with ‘how’ and ‘why,’ encourages students to explicitly state their understanding of disease process, uncovering short-cuts and misunderstandings and reinforcing critical thinking [5]. Placing both preclinical and clinical students in the same classroom reduces barriers that often arise between students at different stages of their education. Additionally, holding the conference in the hospital building introduces preclinical students to the clinical environment, while reminding clerkship students that the foundational sciences have a place in clinical practice.

Prior work has shown that students benefit from CPGR, but the single-institution nature of that report raises questions about replicability [3]. Here, we report quantitative data on the medical student experience after CPGR at two other medical schools. While inspired by the original format, these new curricular offerings were run by different faculties, with attention to local curricular details that required some modification of the program structure. This multi-institutional study allows us to comment on the unique and shared experiences of students and faculty who attend and run CPGR, with lessons for those planning to implement similar sessions at their own institutions. We hypothesized that beneficial and positive experience for students attributable to CPGR at different institutions could be achieved, while allowing some flexibility to accommodate institutional curricular and logistical considerations.

## Methods

### Local CPGR structures

The original format of CPGR involved three faculty members: a discussion facilitator, a concept map developer, and a content expert. The discussion facilitator and concept map developer were senior faculty members and played recurring roles in the conference. The content expert was an invited faculty member on a rotating basis. Case-based discussions occurred in the large group setting with active faculty facilitation to maintain focus, without the use of small groups [3]. Please visit https://vimeo.com/user97225148 to view videos reviewing CPGR techniques. Cases are selected by facilitators to cover a variety of organ systems over the course of the year.

In 2014, a CPGR conference was established at Columbia University College of Physicians and Surgeons (P&S). In contrast to BIDMC, the conference at P&S was led by a third-year medicine resident and a chief resident, and then subsequently by two chief residents. A content expert was still employed, but the brief presentation by the content expert at the end of the conference [3] was omitted and content experts were instead selected to be clinical experts, rather than basic science experts. Facilitators developed a consensus concept map prior to all sessions in private, which they then reconstructed in real-time during the sessions as students pieced together the case. A complete concept map was sent to attendees after the session.

In 2015, Medical University of South Carolina (MUSC) began holding CPGR. The internal medicine residency program director and a second faculty member led the discussion. No content expert was employed, nor was there a formal presentation at the end of the session. In the place of near-peer teaching by the student presenter at P&S, MUSC employed a Think-Pair-Share technique that prompted peer teaching of key concepts [6]. Concept maps were incorporated into the session, developed by a faculty member to reflect the students’ discussion and thinking in real time.

In addition to the delineated difference in CPGR structure, there are differences in the underlying curriculum at the two schools. P&S has a pass-fail, organ system based, one-and-a-half-year preclinical curriculum, while MUSC has a move traditional two-year preclinical curriculum.

The approximate class size is at 150 students at P&S and 170 students at MUSC.

### Study design and implementation

We developed a survey instrument with three domains: perceived benefits of attending CPGR, impact of using concept maps during the CPGR sessions, and value of the mixed-learner environment. These domains included several items that corresponded to themes previously identified by focus groups of students participating in CPGR at BIDMC [3].

We pre-tested the survey with three individuals who had attended CPGR at P&S as medical students but had already graduated and were, thus, ineligible for study inclusion. No changes were suggested based on the respondents’ ‘think out loud’ responses to the survey during pre-testing. We then pilot tested the survey with 5 individuals from the target population. Again, no changes were suggested by pilot testing and all questions showed some degree of differentiation.

All individuals who had attended at least one CPGR session in the proceeding academic year (2015–2016) were eligible to participate in the study. Participation was solicited in April and May 2016 at P&S and May through July 2016 at MUSC. Eligible students received an email link to a survey in Qualtrics and up to two reminder emails.

The survey primarily comprised questions that utilized a five-point Likert scale. For survey items for which the distribution of student responses differed, statistical significance was assessed by Mann-Whitney U tests. Analysis was conducted using Stata Statistical Software: Release 13 (College Station, TX: StataCorp LP). This study was deemed exempt by our Institutional Review Board.

## Results

At P&S, 57 of 100 (57%) eligible students took the survey. At MUSC, 56 of 84 (67%) eligible students. This resulted in an overall response rate of 61%.

### Demographics of attendees

The P&S audience was nearly evenly split among first year students, students in core clerkships, and those in their final year of medical school. By contrast, the MUSC audience was more heavily weighted towards preclinical students (Table 1). The vast majority of students had started attending CPGR during their preclinical years including many who were clinical students at P&S the time of the survey. Nearly one-third of subjects had been to five or more sessions, but those who had attended only one or two sessions were also represented in the survey, comprising 15.9% and 25.7% of the sample, respectively.

**Table 1.** Demographics

Year in School, n (%)	P&S (n = 57)	MUSC (n = 56)	Total (n = 113)
Preclinical	24 (42.1)	44 (78.6)	66 (58.4)
Core Clerkship Year	18 (31.6)	5 (8.9)	23 (20.4)
Post-Core Clerkship	15 (26.3)	7 (12.5)	22 (19.5)
First Attended, n (%)			
Preclinical	50 (87.7)	49 (87.5)	99 (87.6)
Core Clerkship Year	6 (10.5)	6 (10.7)	12 (10.6)
Post-Core Clerkship	1 (1.8)	1 (1.8)	2 (1.8)
Number of Sessions Attended, n (%)			
1	8 (14.0)	10 (17.9)	18 (15.9)
2	10 (17.5)	19 (33.9)	29 (25.7)
3	9 (15.8)	9 (16.1)	18 (15.9)
4	10 (17.5)	5 (8.9)	15 (13.3)
5 or more	20 (35.1)	13 (23.2)	33 (29.2)

P&S = Columbia University College of Physicians & Surgeons

MUSC = Medical University of South Carolina

### Benefits of attending CPGR

The vast majority of subjects somewhat or strongly agreed that using physiology made it easier to remember clinical presentation (92.9%) (Table 2). Students also agreed that CPGR was responsible for helping them to understand concepts that they had previously only memorized (87.5%).

**Table 2.** Student responses

Benefits of Attending CPGR, n (%) n = 112	Strongly Disagree	Somewhat Disagree	Neither Agree nor Disagree	Somewhat Agree	Strongly Agree
It is not practical to think about physiology when taking care of patients	58 (51.8)	28 (25.0)	22 (19.6)	3 (2.7)	1 (0.9)
Once I understand the physiology underlying a disease, it is easier to understand how that disease presents	1 (0.9)	1 (0.9)	6 (5.4)	45 (40.2)	59 (52.7)
Once I understand the physiology underlying a disease, it is easier to understand how to best treat it	1 (0.9)	1 (0.9)	22 (19.6)	39 (34.8)	49 (43.8)
Employing physiology to understand clinical scenarios makes complex patients less intimidating	1 (0.9)	2 (1.8)	18 (16.1)	48 (42.9)	43 (38.4)
By the end of CPGR, I understand some concepts that I had previously simply memorized	0 (0)	2 (1.8)	12 (10.7)	56 (50.0)	42 (37.5)
Concept Maps, n (%) n = 103	Strongly Disagree	Somewhat Disagree	Neither Agree nor Disagree	Somewhat Agree	Strongly Agree
Concept maps help me relate clinical presentations to underlying physiology	0 (0)	3 (2.9)	24 (23.3)	49 (47.6)	27 (26.2)
Concept maps help me understand how diseases work	0 (0)	5 (4.9)	27 (26.2)	42 (40.8)	29 (28.2)
Mixed Learner Environment, n (%) n = 98	Strongly Disagree	Somewhat Disagree	Neither Agree nor Disagree	Somewhat Agree	Strongly Agree
It is worthwhile to see how people from different class years approach clinical problems differently	0 (0)	1 (1.0)	6 (6.1)	41 (41.8)	50 (51.0)
CPGR helps me realize that my fellow students are a great resource	0 (0)	1 (1.0)	12 (12.2)	33 (33.7)	52 (53.1)

Responses are combined across schools. Please see supplemental appendix for results separated by school.

While most students (78.6%) agreed that CPGR facilitated understanding of how to treat patients, there was a significant difference (p < 0.001) in the distribution of responses between preclinical and clinical students. Preclinical students more commonly selected a neutral response, while the vast majority (91.1%) of clinical students agreed that CPGR influenced patient care.

Compared to students at P&S, significantly more students at MUSC identified the teaching modality in CPGR to be distinctive from the rest of their curriculum (69.6% vs 42.9% respectively, p = 0.002).

The full panel of response separated by school can be found in Supplemental Appendix Tables S1 and S2.

### Impact of concept maps

Most students (73.9%) agreed that concept maps helped relate clinical presentations to underlying physiology. Furthermore, most students concurred that concept maps were helpful for understanding diseases (68.9%). Nearly half of students at P&S reported making concept maps outside of CPGR (45.3%), but few did so at MUSC (7.1%).

### Impact of the mixed-learner environment

The vast majority of both clinical (92.5%) and preclinical students (93.1%) were in agreement that a mixed-learning environment was worthwhile. Additionally, 86.7% students felt that CPGR helped them realize their fellow students were a great resource for knowledge, skills, and perspectives.

When clinical students reflected on the impact of having the preclinical students participate in the same case discussion, nearly two-thirds agreed that the preclinical students contributed details of physiology that enhanced their understanding (24/39 or 61.5%). Nearly all (52/57 or 91.2%) of the preclinical students found it exciting to see how much the clinical students knew.

## Discussion

We report on the novel introduction of CPGR by different faculties at two institutions. Our principal finding is that this novel, vertically integrated, interactive teaching conference provides benefits that are replicable and not dependent on specific facilitators. We also note that changes to conference format, in order to best meet institutional needs and resources, did not substantially change the student experience. Our results demonstrate that the benefits of CPGR are preserved across institutions in different geographic regions of the United States and different curricula.

Subject responses, our own experience with CGPR, and learning theory provide insight into why CPGR could be replicable across institutions. The use of concept maps prompts both students and faculty to using inductive reasoning, rather than memorization, to explain findings and predict appropriate management; the discussion focuses on employing concepts of human biology to explain symptoms, physical exam findings and laboratory results rather than on recall of illness scripts [7]. The vertically integrated classroom allows students to utilize the freshness of physiology principles from the preclinical students and greater familiarity with clinical terminology from the clinical students to bridge the gap between the classroom and the hospital. The benefits were bilateral, and not simply a matter of transmission from clinical to pre-clinical students.

As the discussion in CPGR is grounded in the Socratic method, many, if not most, of the connections between presentation and management and underlying physiology are explained by a student to other members of the audience. A student presenter at P&S and think-pair-share activities at MUSC further emphasized peer and near-peer explanation. As such, without requiring any preparation on the part of audience members, the style of the discussion and the mixed-learner environment capture many of the benefits of near-peer teaching. Near-peer teaching has shown to have several distinct benefits including improving communication skills [8], gaining experience and confidence for the future role in academic practice [2,9–15], increased student satisfaction [2,9–11], and providing role models for more junior students [15–17]. Concordant with our own experience leading CPGR sessions, several studies have shown that near-peer teaching can match or even exceed the performance of faculty teaching, which may relate to near-peers’ ability to more clearly communicate information due to greater congruence of context, understanding, and language [18–21]. Thus, while not representing pure near-peer teaching, by focusing on student explanations and presenters, CPGR serves as a venue to emphasize the potential for collaborative learning and forming a community of learners in the learning and practice of medicine.

CPGR have been instituted as a voluntary extracurricular activity; consistent attendance by students indicates that they value the experience. While it is not the primary target of the conference, our findings suggest that CPGR affords an opportunity to smooth the transition from the preclinical to the clinical experience and excites students about these connections and the experiences of their more senior colleagues. As such, the focus on other students as resources emphasizes the importance of an intellectual and peer and near-peer community support network that may not exist in other places in the formal medical school curriculum. In this context, it is possible that in addition to improving vertically integrated knowledge and diagnostic reasoning, CPGR may serve to strengthen a sense of community and wellness. Investigating this potential benefit should be evaluated with future research.

We found similar benefits across the two institutions included in this study despite some key differences in the way sessions were run, as well as differences in the medical schools’ underlying curricula. Sessions at P&S were run by a senior internal medicine resident and chief residents, with the use of a different clinical content expert for each session, while at MUSC, more senior faculty ran the sessions without the help of content experts. As such, compared to the original conference at BIDMC, one site differed in the level of seniority of faculty facilitators, while the other site differed in the use of a content expert. Additionally, MUSC employed intermittent small group break outs, using small group discussions or the Think-Pair-Share technique, unlike the original conference at BIDMC and the P&S sessions. The length of the preclinical curriculum and the frequency with which students encountered this approach to clinical reasoning also differed by site. These variations demonstrate that not only are the benefits of CPGR not specific to the environment at BIDMC or HMS, but that there is flexibility in CPGR sessions to tailor aspects of this conference to local resources and interest, while still preserving the core principles and benefits of CPGR. This was robust to the more traditional two-year preclinical curriculum at MUSC and the integrated one-and-a-half-year curriculum at P&S.

Our results did demonstrate some differences seen between the two sites. A larger proportion of participants at MUSC were preclinical students. Given that students at both sites reported first attending CPGR as preclinical students, and given that the conference at MUSC had been running for less than two years at the time of the survey, we suspect that this reflects insufficient time for students to age into the clinical curriculum rather than a fundamental difference in the attractiveness or utility of the conference for clinical students at this site. This is supported by the fact that the clinical students at MUSC who responded to the survey, had similar responses to the clinical students at P&S as well as to the preclinical students at MUSC.

While nearly half of P&S students reported making concept maps outside of CPGR, few students from MUSC did so. It is unclear if this reflects the impact of fewer clinical students in the MUSC cohort, a greater emphasis placed on building the concept map at P&S, or, perhaps, the presence of other similar teaching methods elsewhere in the P&S curriculum. Indeed, the P&S students were less likely to report that CPGR was distinctive from other modalities in the curriculum, implying they may have had more practice using concept maps in other settings. For the P&S cohort, use of concept maps outside of CPGR reflects a higher order, behavioral outcome [22].

Our study has limitations. First, the benefits attributed to CPGR were determined by self-report and learner perception, not direct measurement. As such, it is possible that students overestimated the benefits they experienced. It is also possible that their responses were affected by a social acceptability or positive response bias, particularly because survey participation was solicited by the faculty running the CPGR sessions at each institution. While we tried to avoid bias in the instrument, participants were likely able to anticipate which responses were consistent with the aims of and learning theory behind CGPR. Furthermore, students with negative impressions of CPGR may have chosen not to respond to the survey, though surveys were anonymous and asynchronous to minimize the risk of this bias. An additional potential source of bias would be the non-random attendance at sessions, as all CPGR sessions are voluntary and student attendance was not mandated. However, while this would limit generalizability to all medical students, it would not be expected to compromise the validity of our findings for those who chose to attend. Any extracurricular conference is likely to face this limitation. Finally, CPGR and our surveys were conducted in the context of the American medical training model. As such, we cannot know the impact for students in other training models. Despite this limitation, we suspect that the benefits may intrinsic to the acquisition of medical knowledge and clinical reasoning than the underlying training model, which is consistent with our finding of preserved benefit with varied curricula within the American model.

## Conclusion

We demonstrate that the previously shown benefits of CPGR are replicable at two other medical schools in the United States, with different facilitators leading sessions at each site. By focusing on the utilization of a mixed-learner environment and concept maps as a visual demonstration of inductive reasoning and vertical integration, a variable composition of facilitators and session structure can still capture the benefits of CPGR. Such flexibility can allow for the adoption of this teaching format across different resource constraints, while preserving its core tenants and the resultant benefit to students.

## Supplementary Material

Supplemental Material

## Data Availability

ur raw data has been uploaded to Harvard Dataverse and can be accessed at https://doi.org/10.7910/DVN/YUCN8Y
