# Supplementary material for: Building upon the foundational science curriculum with physiology-based grand rounds: a multi-institutional program evaluation
Source: Med Educ Online. 2021 Jun 11;26(1):1937908. doi: 10.1080/10872981.2021.1937908 (PMC8204959; doi:10.1080/10872981.2021.1937908)
Supplement: Supplemental Material [file ZMEO_A_1937908_SM0356.zip › supplementary/Appendix_Table1_PS_Only.docx]

Appendix Table 1. P&S Student responses

| Benefits of Attending CPGR, n (%)  n=56 | | Strongly Disagree | Somewhat Disagree | Neither Agree nor Disagree | Somewhat Agree | Strongly  Agree |
| --- | --- | --- | --- | --- | --- | --- |
|  | It is not practical to think about physiology when taking care of patients | 46 (82.1) | 8 (14.3) | 2 (3.6) | 0 (0) | 0 (0) |
|  | Once I understand the physiology underlying a disease, it is easier to understand how that disease presents | 0 (0) | 1 (1.8) | 0 (0) | 11 (19.6) | 44 (78.6) |
|  | Once I understand the physiology underlying a disease, it is easier to understand how to best treat it | 0 (0) | 0 (0) | 2 (3.6) | 15 (26.8) | 39 (69.6) |
|  | Employing physiology to understand clinical scenarios makes complex patients less intimidating | 0 (0) | 1 (1.8) | 6 (10.7) | 17 (30.4) | 32 (57.1) |
|  | By the end of CPGR, I understand some concepts that I had previously simply memorized | 0 (0) | 1 (1.8) | 5 (8.9) | 25 (44.6) | 25 (44.6) |
| Concept Maps, n (%)  n=53 | | **Strongly Disagree** | **Somewhat Disagree** | **Neither Agree nor Disagree** | **Somewhat Agree** | **Strongly**  **Agree** |
|  | Concept maps help me relate clinical presentations to underlying physiology | 0 (0) | 1 (1.9) | 8 (15.1) | 23 (43.4) | 21 (39.6) |
|  | Concept maps help me understand how diseases work | 0 (0) | 1 (1.9) | 11 (20.8) | 18 (34.0) | 23 (43.4) |
|  | Concept maps help me remember how diseases work | 0 (0) | 3 (5.7) | 11 (20.8) | 23 (43.4) | 16 (30.2) |
| Mixed Learner Environment, n (%)  n=51 | | **Strongly Disagree** | **Somewhat Disagree** | **Neither Agree nor Disagree** | **Somewhat Agree** | **Strongly**  **Agree** |
|  | It is worthwhile to see how people from different class years approach clinical problems differently | 0 (0) | 1 (2.0) | 2 (3.9) | 12 (23.5) | 36 (70.6) |
|  | CPGR helps me realize that my fellow students are a great resource | 0 (0) | 1 (2.0) | 9 (17.7) | 13 (25.5) | 28 (54.9) |
