# Supplementary material for: Building upon the foundational science curriculum with physiology-based grand rounds: a multi-institutional program evaluation
Source: Med Educ Online. 2021 Jun 11;26(1):1937908. doi: 10.1080/10872981.2021.1937908 (PMC8204959; doi:10.1080/10872981.2021.1937908)
Supplement: Supplemental Material [file ZMEO_A_1937908_SM0356.zip › supplementary/Appendix_Table2_MUSC_Only_.docx]

Appendix Table 2. MUSC Student responses

| Benefits of Attending CPGR, n (%)  n=56 | | Strongly Disagree | Somewhat Disagree | Neither Agree nor Disagree | Somewhat Agree | Strongly  Agree |
| --- | --- | --- | --- | --- | --- | --- |
|  | It is not practical to think about physiology when taking care of patients | 12 (21.4) | 20 (35.7) | 20 (35.7) | 3 (5.4) | 1 (1.8) |
|  | Once I understand the physiology underlying a disease, it is easier to understand how that disease presents | 1 (1.8) | 0 (0) | 6 (10.7) | 34 (60.7) | 15 (26.8) |
|  | Once I understand the physiology underlying a disease, it is easier to understand how to best treat it | 1 (1.8) | 1 (1.8) | 20 (35.7) | 24 (42.9) | 10 (17.9) |
|  | Employing physiology to understand clinical scenarios makes complex patients less intimidating | 1 (1.8) | 1 (1.8) | 12 (21.4) | 31 (55.4) | 11 (19.6) |
|  | By the end of CPGR, I understand some concepts that I had previously simply memorized | 0 (0) | 1 (1.8) | 7 (12.5) | 31 (55.4) | 17 (30.4) |
| Concept Maps, n (%)  n=50 | | **Strongly Disagree** | **Somewhat Disagree** | **Neither Agree nor Disagree** | **Somewhat Agree** | **Strongly**  **Agree** |
|  | Concept maps help me relate clinical presentations to underlying physiology | 0 (0) | 2 (4.0) | 16 (32.0) | 26 (52.0) | 6 (12.0) |
|  | Concept maps help me understand how diseases work | 0 (0) | 4 (8.0) | 16 (32.0) | 24 (48.0) | 6 (12.0) |
|  |  |  |  |  |  |  |
| Mixed Learner Environment, n (%)  n=47 | | **Strongly Disagree** | **Somewhat Disagree** | **Neither Agree nor Disagree** | **Somewhat Agree** | **Strongly**  **Agree** |
|  | It is worthwhile to see how people from different class years approach clinical problems differently | 0 (0) | 0 (0) | 4 (8.5) | 29 (61.7) | 14 (29.8) |
|  | CPGR helps me realize that my fellow students are a great resource | 0 (0) | 0 (0) | 3 (6.4) | 20 (42.6) | 24 (51.1) |
